# Optimized in vivo two-photon imaging reveals the essential role of the contralateral eye in functional optic nerve regeneration in zebrafish larvae

**DOI:** 10.1186/s40662-025-00447-z

**Published:** 2025-08-25

**Authors:** Baoguo Shen, Hongyuan Wei, Yuan Wen, Yuan Geng, Tonghe Yang, Ziwen Chen, Siyu Dong, Yuwan Gao, Ting Li, Lanfang Sun, Bin Xie, Wentao Yan, Yikui Zhang, Wencan Wu

**Affiliations:** 1 State Key Laboratory of Eye Health, Eye Hospital, Wenzhou Medical University, Wenzhou, China; 2 Zhejiang Key Laboratory of Key Technologies for Visual Pathway Reconstruction, Eye Hospital, Wenzhou Medical University, Wenzhou, China; 3 Oujiang Laboratory (Zhejiang Lab for Regenerative Medicine, Vision and Brain Health), Wenzhou, Zhejiang China

**Keywords:** Zebrafish larvae, Optic nerve regeneration, In vivo imaging, Behavioral recovery

## Abstract

**Background:**

The visual pathway, consisting of the eye, optic nerve, and brain, serves as a valuable model for studying neural regeneration. The exceptional regenerative capacity of the zebrafish visual system enables detailed investigation of neural repair mechanisms in vivo. Although the transparency of zebrafish larvae permits real-time imaging of axonal regeneration following transection, previous methodological limitations such as pigment interference and suboptimal imaging protocols have hindered high-resolution analyses of structural recovery and cellular interaction throughout the entire visual pathway after optic nerve injury. This study aimed to overcome these barriers and enable comprehensive assessment of visual pathway regeneration.

**Methods:**

In this study, we dissect the regenerative processes underlying structural recovery and cellular interplay across the entire visual pathway in larval zebrafish with an optic nerve transection model, using two-photon imaging and optokinetic response assays. Data were analyzed via multi-factorial ANOVA, unpaired *t-tests*, or Welch’s *t-test*.

**Results:**

We developed a longitudinal imaging platform by integrating two-photon microscopy (930 nm excitation), pigment suppression with phenylthiourea (PTU), and multi-axis positioning to observe visual pathway regeneration in vivo in zebrafish larvae at cellular resolution. This system enabled high-resolution imaging of the entire visual pathway, capturing the dynamics of green fluorescent protein (GFP)-labeled retinal ganglion cell (RGC) axons, optic nerve projections, and tectal reinnervation following optic nerve transection. Notably, enucleation of the contralateral eye resulted in aberrant optic nerve regrowth and impaired visual recovery after transection, indicating that guidance cues from the contralateral eye were essential for successful functional optic nerve regeneration. Additionally, the optimized two-photon imaging protocol allowed direct in vivo visualization of cellular interactions, revealing the rapid recruitment of DsRed-labeled neutrophils to the injured retina, optic nerve, and tectum during the repair process in double-transgenic *Tg*(*lyz:DsRed*); *Tg*(*isl2b.2:Gal4-VP16; myl7:EGFP); Tg(4XnrUAS:GFP*) larvae.

**Conclusions:**

Our optimized imaging platform visualizes the entire visual pathway and cell interactions during regeneration, revealing contralateral eye is essential for functional recovery following optic nerve transection. Combined with multi-omics and calcium imaging, this approach potentially provides a powerful platform to decipher the cellular and molecular mechanisms of zebrafish eye-brain pathway reconstruction and offers insights into therapeutic targets for human optic neuropathies.

**Supplementary Information:**

The online version contains supplementary material available at 10.1186/s40662-025-00447-z.

## Background

Optic neuropathies including glaucoma, traumatic injury, ischemic damage, and optic neuritis, are the leading causes of irreversible blindness worldwide [1]; however, effective treatments for optic nerve repair remain elusive despite decades of research [2]. This clinical challenge poses substantial socioeconomic costs and highlights the urgent need for regenerative therapies that are translatable for clinical use.

Traditional mammalian models, such as mice, present inherent limitations for studying optic nerve regeneration due to the optic nerve's inability to regenerate, embryonic opacity, complex ocular anatomy, and the labor-intensive nature of tissue processing, which collectively impede real-time monitoring of injury responses [1, 3, 4]. In contrast, zebrafish larvae offer distinct experimental advantages: their transparency facilitates noninvasive imaging, and the conservation of glial cell types, including microglia, and human-like immune responses involving neutrophils and macrophages enable dynamic in vivo monitoring of inflammation and regeneration [5–7].

In this study, we used optimized two-photon microscopy with near-infrared excitation (930 nm) and high-resolution Z-stack imaging (2 μm intervals) to investigate optic nerve regeneration in zebrafish larvae following transection. This approach overcomes the technical limitations of mammalian models and enables precise visualization of axonal regeneration and cell–cell interactions at subcellular resolution.

Our platform provides a scalable system for screening regenerative therapies, advancing the understanding of their mechanisms and accelerating therapeutic discovery. By correlating structural regeneration with functional recovery, as assessed by optokinetic response (OKR), our study establishes a framework for identifying evolutionarily conserved mechanisms of optic nerve repair.

## Methods

### Animals

*Tg(Lyz:Dsred)* zebrafish were purchased from the China Zebrafish Resource Center (CZRC, ID: CZ59, China). *Tg(isl2b.2:Gal4-VP16,myl7:EGFP)*;*Tg(4XnrUAS:GFP)* were kindly provided by Professor Jiulin Du (Institute of Neuroscience, State Key Laboratory of Neuroscience, Center for Excellence in Brain Science and Intelligence Technology, Chinese Academy of Sciences, Shanghai, China). Zebrafish were maintained under standard conditions (14 h light/10 h dark cycle, 28.5 °C), with embryos [0–7 days after fertilization (dpf)] reared in E3 medium (5 mM NaCl, 0.17 mM KCl, 0.33 mM CaCl_2_, 0.33 mM MgSO_4_). They were fed in the morning and evening and housed at the Eye Hospital of Wenzhou Medical University. To suppress melanocyte pigmentation, zebrafish larvae were maintained in E3 medium containing 0.2 mM PTU (Sigma-Aldrich, USA) at 28.5 °C starting at 1 dpf.

### Hematoxylin and eosin (H&E) staining

The larvae (5 dpf) were fixed in Bouin’s solution (Phygene, China) at 4 °C for 12 h then rinsed and dehydrated through a series of ethanol, xylene, and paraffin solutions using a HistoCore PEARL processor (Leica, Germany). Following dehydration, the larvae were embedded in paraffin with their heads facing downward and sectioned at 3 μm thickness using a HistoCore BIOCUT (Leica, Germany).

The sections were dried at 65 °C for 1 h. Dewaxing and H&E staining were performed using an Autostainer XL (Leica, Germany). Tissue sections were deparaffinized in xylene for 8 min, rehydrated through graded ethanol (100% for 6 min, 70% for 2 min), and rinsed in water for 2 min. Hematoxylin staining was performed for 3 min, followed by differentiation (45 s), bluing (1 min), and eosin counterstaining (1 min). Slides were dehydrated in ethanol (95% for 2 min, 100% for 4 min) and cleared in xylene for 4 min. The images were acquired and analyzed using the 3D Pannoramic Scanner Software (3DHISTECH, Hungary).

### Larval eye enucleation

Larvae were embedded with the dorsal side up in 1.5% low-melting-point agarose (Sigma-Aldrich, USA) prepared in E3 medium. Using a SZ61TR microscope (Olympus, Japan), the right eye was excised at the proximal exit site with sharpened insect pins. After excision, larvae were released from the agarose and allowed to recover for 1 h in Ringer’s solution (38.7 mM NaCl, 1.0 mM KCl, 1.7 mM HEPES, 2.4 mM CaCl_2_, pH 7.2) supplemented with 0.2 mM PTU. Following recovery, larvae were transferred to E3 medium containing 0.2 mM PTU. After 24 h, larval viability was confirmed, and the optic nerve was transected for subsequent imaging or OKR experiments.

### Optic nerve transection

Optic nerve transection was performed on zebrafish larvae at 5 dpf, as previously described [8]. In brief, larvae were immobilized ventrally in a petri dish containing 1.5% low-melting-point agarose and visualized using a SZX16 fluorescence microscope (Olympus, Japan). The left optic nerve was transected proximally to its exit from the eye using sharpened insect pins. Partial transections were confirmed by the persistence of GFP-positive fibers. Injured larvae were released from the agarose and allowed to recover for 1 h in Ringer’s solution supplemented with 0.2 mM PTU, before being returned to E3 medium containing 0.2 mM PTU. At 24 h post-injury (hpi), lesion completeness was assessed by fluorescence: larvae with no detectable GFP signal in both the optic nerve and tectum were classified as complete transections and maintained in PTU-E3 medium for downstream fixation or live imaging. Larvae retaining GFP fluorescence in both the optic nerve and tectum were classified as partial transections.

For the transection experiments, each trial was conducted using 10 zebrafish larvae, from which 3 to 4 larvae with confirmed complete optic nerve transection were selected for further analysis.

### Two-photon imaging

As previously described [9], zebrafish larvae at 5 dpf were mounted dorsally in 1.5% low-melting agarose (prepared in E3 medium) in 35 mm glass bottom dishes (NEST Biotechnology, China). To prevent desiccation and ensure immobilization, larvae were maintained in E3 medium containing 0.2 mM PTU and 0.002% tricaine (Sigma-Aldrich, USA). Two-photon imaging was performed on transgenic zebrafish lines *[Tg(isl2b.2:Gal4-VP16, myl7:EGFP); Tg(4XnrUAS:GFP)]* using a multiphoton laser-scanning microscope (LSM 880 NLO with AiryScan; Carl Zeiss, Germany). GFP was excited using a Chameleon Ultra II IR laser (Coherent, USA), with the wavelength and intensity controlled using ZEISS (Zen Blue 3.1) software. The laser intensities were calibrated to 90, 85, and 86 mW for wavelengths of 800, 930, and 1,040 nm, respectively. Imaging was performed using an Objective W Plan-Apochromat 20 × (NA 1.05; Zeiss, Germany) at a resolution of 1,024 × 1,024 pixels. Z-plane adjustments were made at intervals of 2, 6, and 12 µm, and Zoom = 0.7 was applied as necessary to optimize the imaging depth of the volumetric structures. To enlarge the optic nerve region, a zoom factor of 1.5 was applied.

Further imaging was performed on double-transgenic zebrafish lines *[Tg(Lyz:DsRed)* and *Tg(Isl2b.2:Gal4-VP16, myl7:EGFP); Tg(4XnrUAS:GFP)]* using an FVMPE-RS multiphoton microscope (Olympus, Japan). GFP was excited using a Mai Tai HP DeepSee IR laser (Spectra-Physics, USA) at 920 nm (44.7 mW, 3% of 1.49 W total power), and DsRed was excited using an Insight X3 IR laser (Spectra-Physics, USA) at 1,100 nm (51.2 mW, 4% of 1.28 W total power). The imaging was performed using a 25 × water-immersion objective (NA 1.05; Olympus, Japan) at a resolution of 1,024 × 1,024 pixels. Z-plane adjustments were made at 2 µm intervals to optimize the imaging depth for visualizing the volumetric structures. All images were processed using Imaris software (Oxford Instruments, UK). Quantitative analyses of fluorescence intensity were performed using ImageJ (https://imagej.net/ij/). For the imaging experiments, each trial was performed using 3 to 6 zebrafish larvae.

### OKR assay

OKR assays were performed as previously described [10]. Zebrafish larvae were collected by pipette with minimal water, briefly embedded in 1% low-melting-point agarose, and centrally positioned in 35 mm glass-bottom culture dishes. Their posture was then adjusted with fine forceps to avoid tissue injury. Upon agarose solidification, the culture dishes were carefully overlaid with E3 embryo medium. Subsequently, triangular apertures were precisely excised in the agarose to ensure unrestricted ocular motility. The preparations were then transferred to the OKR apparatus (ViewPoint, France). The stimulus parameters (i.e., contrast, color, spatial frequency, and angular velocity) were controlled using software. A 5-min training stimulus (100% contrast, black/white stripes) was provided before the experiments to stabilize tracking behaviors. Spatial frequencies were incrementally increased during testing, and eye movements were quantified by manual counting at 1-min intervals.

After the experiment, the larvae were gently extricated from the agarose and returned to the fresh system water. Eye rotations per minute were manually counted and analyzed using video recordings.

To determine the optimal stripe pattern for the OKR assay in zebrafish larvae, uninjured larvae were tested with grating frequencies of 0, 5, 10, and 20 stripes. A sample size of n = 6 larvae per group was used for each condition. In experiments involving PTU treatment, zebrafish larvae were exposed to PTU until 5 dpf. Subsequently, larvae were transferred to E3 medium and allowed to recover eye pigmentation over a period of 2.5 days. Following this recovery phase, OKR assays were conducted. Sample sizes were n = 9 larvae for the non-PTU control group and n = 10 for the PTU-treated group.

To minimize visual input from the contralateral eye, we conducted a monocular occlusion experiment. Healthy larvae were tested with monocular occlusion (n = 16) and without monocular occlusion (n = 16). In a subset of experiments, the uninjured eye was occluded with tin foil. For injured larvae, experiments were performed both with and without covering the uninjured eye. For the group without covering the uninjured eye, the sample sizes were as follows: uninjured (n = 9), 24 hpi (n = 6), and 72 hpi (n = 8). For larvae with the uninjured eye covered, the sample sizes were uninjured (n = 9), 24 hpi (n = 8), and 72 hpi (n = 8).

For enucleation experiments, viable larvae were selected for the OKR assay at 24 h post-enucleation. The sample sizes for OKR assessments were as follows: baseline (n = 7 for 8 dpf larvae, n = 12 for 5 dpf larvae), post-enucleation (n = 6 larvae), and post-contralateral optic nerve injury (n = 3 larvae).

### Statistical analysis

All statistical analyses were performed using GraphPad Prism (version 9.5.0). One-way analysis of variance (ANOVA), followed by Dunnett’s test, was performed for comparisons among three or more groups. The unpaired *t-test* or Welch’s *t-test* was used for comparing data from two independent groups. Data are expressed as means ± standard error of the mean, and *P* < 0.05 were used to denote statistically significant differences.

## Results

**Fig. 1:**
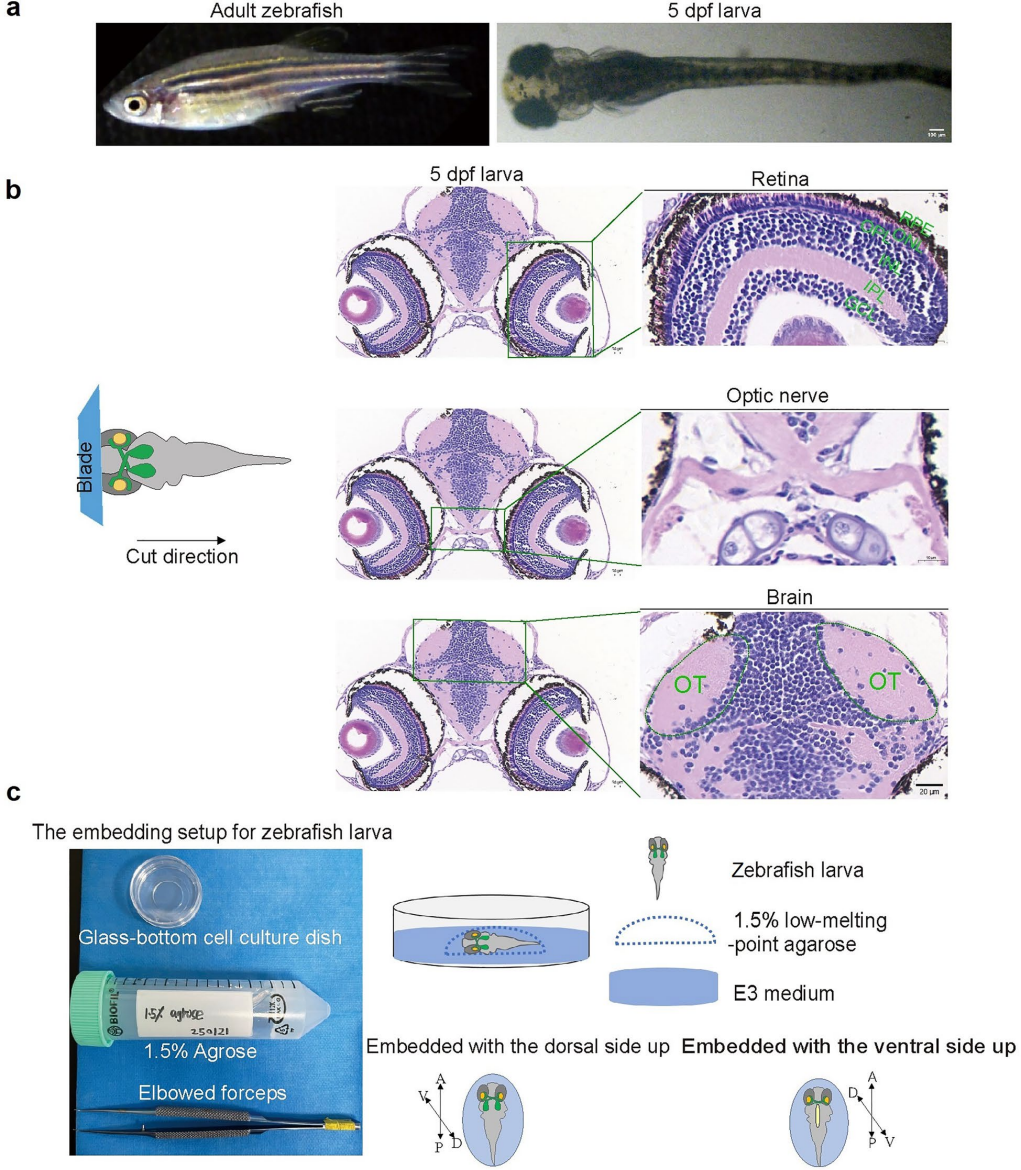
Histological and cellular characterization of zebrafish larval development. **a** Morphological comparison of an adult zebrafish (left) and 5 days post-fertilization (dpf) larva (right). Scale bar: 100 µm. **b** Left: schematic of the paraffin-embedding and sectioning protocols for zebrafish larvae. Right: histological sections highlighting the retina, optic nerve, and brain (green boxes indicate magnified regions). Scale bars: larval overview, 50 µm; retina, 20 µm; optic nerve, 10 µm; brain, 20 µm. **c** Left: key apparatus for larval embedding. Right: schematic of larval zebrafish immobilized in low-melting-point agarose for imaging. The diagram illustrates different embedding orientations. Anterior (A), posterior (P), dorsal (D), and ventral (V) anatomical directions are indicated

Zebrafish larvae exhibit natural transparency throughout their bodies, making them an exemplary model for in vivo imaging studies (Fig. 1a). To confirm the developmental maturity of the visual system at 5 dpf, H&E staining was performed on paraffin-embedded tissue samples, which revealed a developed retina, optic nerve, and brain (Fig. 1b). While the visual system at 5 dpf exhibits many mature characteristics, it is important to note that processes such as optic tectum stratification and myelination are still in progress, indicating that full maturation is not yet complete [11, 12].

**Fig. 2:**
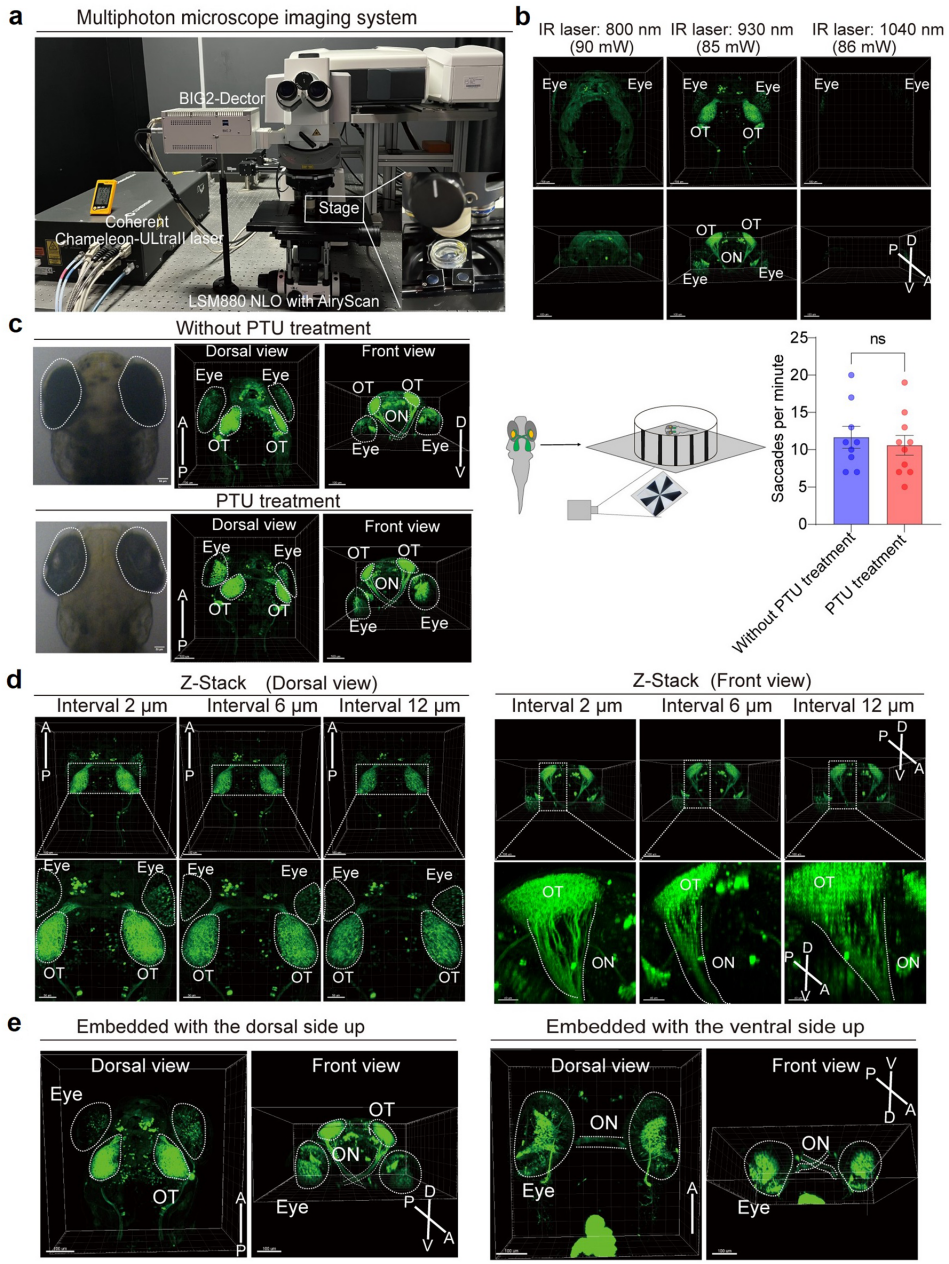
Optimization of two-photon imaging for zebrafish larvae. **a** Multiphoton imaging system. **b** Effect of different laser wavelengths on imaging. Left: dorsal view of the larva at 800 nm wavelength with 90 mW power (top panel) and front view (bottom panel). Middle: dorsal view at 930 nm wavelength with 85 mW power (top panel) and front view (bottom panel). Right: dorsal view at 1,040 nm wavelength with 85 mW power (top panel) and front view (bottom panel). Scale bars: 100 µm. **c** Effects of PTU treatment on imaging and optokinetic response (OKR). Top panel: untreated larvae, showing pigment cells in the eye and brain under light-field microscopy (dorsal view, left panel; front view, right panel). Bottom panel: PTU-treated larva, with reduced pigment cells in the eye and brain (dorsal view, left panel; front view, right panel). Scale bars: 100 µm. Right panel: schematic illustration of zebrafish OKR behavior, along with the statistical analysis of eye movement frequencies before and after PTU treatment. An unpaired *t-test* was employed for statistical analysis. Significance levels were defined as follows: ns indicates no significant difference. The sample sizes were n = 9 larvae in the non-PTU treatment group and n = 10 larvae in the PTU treatment group. **d** Impact of the Z-stack interval on imaging. Left: dorsal view of the larva with varying Z-stack intervals (upper panel). Scale bars: 100 µm. An enlargement of the upper panel (bottom panel). Scale bars: 50 µm. Right: front view of the 3D reconstruction (upper panel). Scale bars: 100 µm. An enlargement of the upper panel (bottom panel). Scale bars: 40 µm. **e** Comparison of different embedding methods for larvae. Left: larva embedded dorsal side up. Right: larva embedded ventral side up. Scale bar: 100 µm. ON, optic nerve; OT, optic tectum. The anterior (A), posterior (P), dorsal (D), and ventral (V) orientations are indicated

For in vivo imaging, zebrafish larvae were immobilized in a glass-bottom dish using 1.5% low-melting-point agarose, with E3 medium added to prevent desiccation (Fig. 1c). Imaging was performed using a Zeiss microscope equipped with a Chameleon Ultra II IR laser system, which can obtain wavelengths between 600 and 1,060 nm (Fig. 2a). To optimize imaging conditions, laser wavelengths of 800, 930, and 1,040 nm were tested at consistent power levels to determine the optimal settings for exciting GFP fluorescence in the retina, optic nerve, and optic tectum in transgenic zebrafish. Our results revealed that 930 nm provided the clearest visualization (Fig. 2b).

A notable challenge in imaging zebrafish larvae is the accumulation of pigment cells, which can impede laser penetration and reduce imaging clarity. To address this, the imaging outcomes were compared between larvae with and without PTU treatment, which inhibits pigment formation [13, 14]. In untreated larvae, although fluorescent signals in the optic nerve and tectum were detectable, retinal signals appeared disorganized because of pigment interference (Fig. 2c). After PTU treatment, retinal fluorescence signals were enhanced despite the presence of residual pigment cells in the eye (Fig. 2c). Previous studies have shown that PTU affects eye development [14]. To determine whether PTU treatment affects the visual behavior in zebrafish, we performed the OKR test in both untreated and PTU-treated zebrafish. In the PTU group, larvae were transferred to E3 medium for recovery after 5 days of PTU exposure. The OKR results revealed no significant difference between PTU-treated and control larvae, indicating that the 0.2 mM PTU concentration used in this study did not affect visual behavior (Fig. 2c).

Z-stack imaging was performed with optimized scanning intervals to achieve comprehensive visualization of the entire visual pathway in zebrafish. Scanning intervals of 2, 6, and 12 µm were tested to determine the optimal setting for clear signal detection. Our results revealed that at a 2 µm interval, the fluorescent signals from the retina and optic tectum were clearly discernible (Fig. 2d). In contrast, at 6 and 12 µm intervals, the signals became progressively blurred, compromising image clarity (Fig. 2d). To further assess the effects of scanning intervals on image quality, 3D reconstruction of the visual pathway was performed. The 2 µm interval yielded sharp and well-defined signals, particularly in the optic tectum and optic nerve regions. However, at the 6 and 12 µm intervals, the clarity of the signals decreased, with notable degradation in the optic nerve region (Fig. 2d). Therefore, 2 µm intervals were chosen for all subsequent experiments.

Furthermore, different embedding orientations were evaluated to optimize imaging. Dorsal-up embedding enabled clear visualization of the fluorescent signals from the retina, optic nerve, and optic tectum (Fig. 2e), whereas dorsal-down embedding allowed detection of the retina and optic nerve but obscured the signals from the optic tectum. Therefore, the dorsal-up orientation was adopted in all subsequent imaging experiments.

**Fig. 3:**
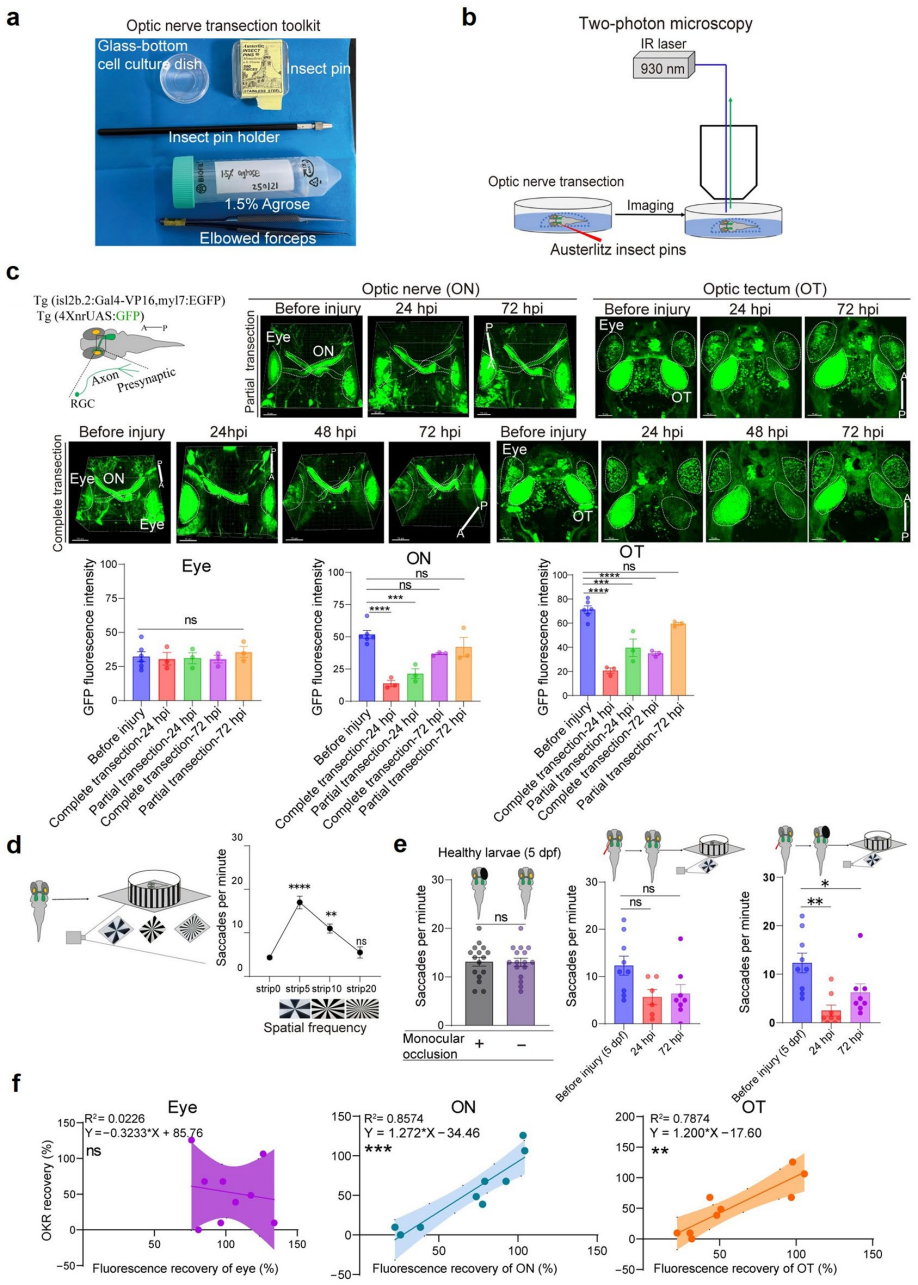
Optic nerve imaging and behavioral assessment following transection in larvae. **a** Larval optic nerve transection setup. **b** Workflow schematic for transection and imaging. **c** Fluorescence dynamics in optic nerve (ON) and optic tectum (OT): top left, simplified transgenic zebrafish diagram; top right, OT changes after partial transection; bottom left, optic nerve changes after complete transection; bottom right, OT changes after complete transection. White dotted traces indicate landmarks of the eye, ON and OT. Quantification of eye, ON, and OT intensity over time (n = 6 for control group, n = 3 for injury group). Orientations: A (anterior), P (posterior), D (dorsal), V (ventral); scale bars, 50 µm or 70 µm . **d** Optokinetic response (OKR) measurement setup: larvae tested at grating frequencies of 0, 5, 10, and 20 stripes (n = 6 each group). **e** OKR responses post-transection with or without monocular occlusion: Left: healthy larvae, with (n = 16) or without (n = 16) monocular occlusion. Middle: larvae without monocular occlusion, at control (n = 9), 24 h post-transection (n = 6), and 72 h post-transection (n = 8). Right: larvae with monocular occlusion, at control (n = 9), 24 h post-transection (n = 8), and 72 h post-transection (n = 8). **f** Correlation analysis of various factors and functional recovery (OKR) during zebrafish visual pathway regeneration. All data were normalized to percentage values relative to pre-injury levels. Each parameter was derived from normalized data pooled across at least three zebrafish per group. Injury time points were aligned with corresponding OKR behavioral measurements to assess recovery dynamics. Correlation analysis was performed using nonlinear regression (curve fitting) in GraphPad Prism. In Figure c–e, statistical analysis was performed using one-way ANOVA with Dunnett’s test. ns, not significant; *P < 0.05; **P < 0.01; ***P < 0.001; ****P < 0.0001

Optic nerve regeneration was investigated by transection of the optic nerve in zebrafish larvae using our established imaging methods. The larvae were immobilized in 1.5% low-melting-point agarose, and the optic nerve of the left eye was transected under a fluorescence microscope using an insect pin (Fig. 3a). Imaging was performed at various time points after injury (Fig. 3b,c). Tg(*isl2b.2:Gal4-VP16; myl7:EGFP); Tg(4XnrUAS:GFP*) larvae, in which the GFP labels the RGCs and their axons [15–17]. Before injury, robust GFP fluorescent signals were observed in the retina, optic nerve, and optic tectum (Fig. 3c). In this study, we employed two distinct injury models: complete transection and partial transection of the unilateral optic nerve. At 24 h post-partial transection, fluorescent signals in the eye remained stable, whereas significant reductions were observed in both the optic nerve and tectum (Fig. 3c). By 72 hpi, fluorescent signals in the eye continued to remain stable, while signals in the optic nerve and tectum had recovered and exhibited no significant difference compared to the contralateral side (Fig. 3c). In the complete transection model, fluorescence in the eye exhibited a pattern similar to that observed in the partial transection model. At 24 hpi, signals in the optic nerve and tectum were nearly undetectable (Fig. 3c). Fluorescence began to reappear in both regions by 48 hpi. By 72 hpi, a marked recovery of fluorescence was evident in both the optic nerve and tectum; however, tectal signals remained significantly weaker than those observed in the partial transection model. This finding is consistent with previous studies [8].

To evaluate the restoration of visual behaviors after optic nerve regeneration, a series of OKR tests were performed on zebrafish larvae at various time points after injury. The OKR assay, a well-established method for assessing visual function in larval zebrafish [10], was performed with the larvae immobilized in low-melting-point agarose, ensuring that the eyes remained exposed to prevent agarose-induced immobilization from interfering with the test (Fig. 3d). Before injury, zebrafish larvae exhibited a gradual increase in eye movement frequency in response to the number of grating stripes, with the peak frequency observed at five stripes (Fig. 3d). Therefore, spatial frequency of five stripes was chosen for all subsequent OKR experiments. Following optic nerve transection, we observed a significant impact on OKR behavior by the contralateral eye (Fig. 3e). However, in healthy larvae, monocular occlusion did not affect their OKR response (Fig. 3e). When the contralateral eye was not covered, we found no significant difference in the number of eye movements between 24 and 72 hpi in the injured zebrafish (Fig. 3e). To minimize the influence of the contralateral eye on the injured side, we covered the contralateral eye with aluminum foil and conducted OKR behavioral analysis (Fig. 3e). At 24 hpi, the OKR frequency was significantly reduced at five strips (Fig. 3e). However, OKR recovery was notable at 72 hpi. These findings highlight the phased recovery of visual function in zebrafish larvae, highlighting their remarkable regenerative capacity and the spatiotemporal dynamics of neural circuit reorganization following optic nerve transection.

The observed recovery pattern suggests that restoration of visual function is a progressive process that aligns with the regeneration timeline of the optic nerve. Correlation analyses showed significant positive associations between functional recovery (as measured by OKR performance) and fluorescence restoration in both the optic nerve and optic tectum, while retinal signal recovery did not correlate with behavioral outcomes (Fig. 3f). Multiple linear regression analysis further confirmed that recovery of fluorescence in both the optic nerve and optic tectum was significantly associated with OKR recovery (Supplementary table).

**Fig. 4:**
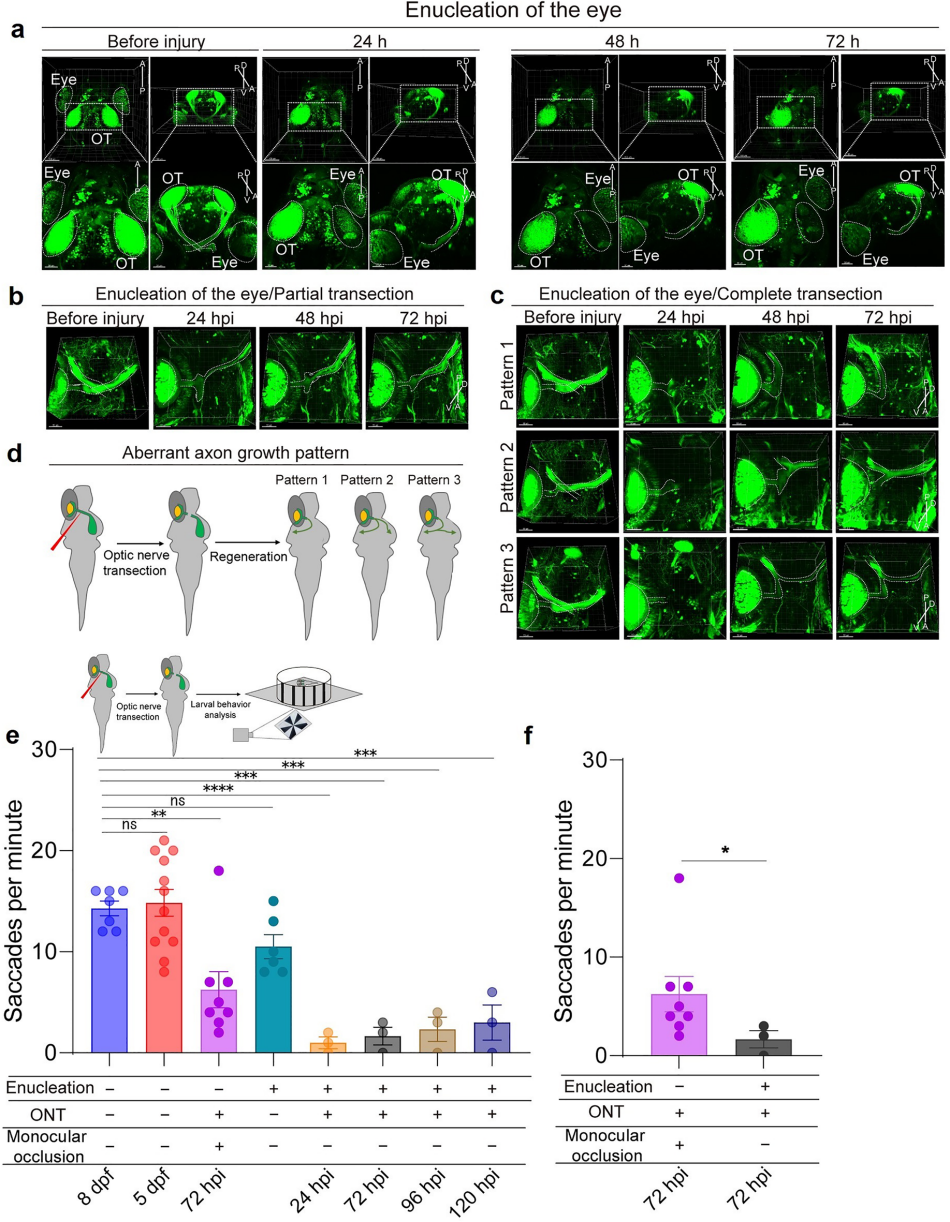
Imaging of the optic nerve and behavioral assay in zebrafish larvae following enucleation. **a** Fluorescence dynamic in optic nerves (ON) and optic tectum (OT) following eyeball removal. Upper panels: larval views (scale bar, 100 µm). Lower panels: enlarged views (scale bar, 50 µm). Orientations: A (anterior), P (posterior), D (dorsal), V (ventral). **b** Optic nerve regeneration after partial transection of the contralateral nerve post-enucleation. White dotted traces indicate landmarks of the eye and ON. Orientations: A (anterior), P (posterior), D (dorsal), V (ventral). Scale bar, 50 µm. **c** Optic nerve regeneration after complete transection of the contralateral nerve post-enucleation. Regeneration categorized into three distinct patterns. White dotted traces indicate landmarks of the eye and ON. Scale bar, 50 µm. **d** Schematic of three patterns of aberrant axon growth following complete axon transection. **e** Top: optokinetic response (OKR) in larvae following unilateral enucleation and contralateral optic nerve transection. Bottom: experimental injury models: (i) no enucleation (8 dpf and 5 dpf larvae), (ii) monocular occlusion with optic nerve transection, (iii) enucleation, and (iv) enucleation with optic nerve transection. Sample sizes: n = 7 (8 dpf), n = 12 (5 dpf), n = 8 (monocular occlusion + ONT), n = 6 (post-enucleation), n = 3 (post-enucleation + ONT). ONT, optic nerve transection. Statistical analysis was performed using one-way ANOVA with Dunnett’s test. Significance levels: ns, not significant, **P < 0.01, ***P < 0.001, ****P < 0.0001. **f** OKR behavior in larvae following unilateral eye injury and enucleation with contralateral optic nerve injury at 72 hpi. Sample sizes: n = 8 pre-enucleation, n = 3 post-enucleation with optic nerve transection. Statistical analysis: Welch’s *t-test*

To further investigate whether the contralateral eye influences regeneration of the injured optic nerve, we enucleated the contralateral eye before transecting the optic nerve of the remaining eye. After enucleation, the ipsilateral optic nerve degenerated without regeneration, and this procedure did not affect fluorescent signals or visual function in the remaining eye (Fig. 4a, e).

When partial optic nerve transection was performed after contralateral enucleation, robust regeneration of the optic nerve was observed, with the process completed within 72 h (Fig. 4b). This suggests that residual ipsilateral axons provide guidance cues that promote organized axon regeneration and reconnection between the eye and the brain. This observation was consistent with findings from a previous study [8].

In contrast, complete optic nerve transection following contralateral enucleation resulted in disordered optic nerve regeneration. Aberrant axon growth patterns were classified into the following categories: Pattern 1, axons projecting into the ipsilateral brain region; Pattern 2, axons projecting into both brain hemispheres; and Pattern 3, axons initially growing into the ipsilateral brain and then crossing over to the contralateral side (Fig. 4c, d).

To more accurately evaluate the extent of optic nerve regeneration, we compared the OKR of 72 hpi larvae with that of uninjured 8 dpf larvae, our findings confirm that, at 72 hpi, optic nerve regeneration in larvae is insufficient to achieve complete functional recovery (Fig. 4e). This result aligns with previous reports in adult zebrafish, where full functional recovery typically requires 2–4 weeks [18–21]. Moreover, our comparison revealed a significant delay in OKR recovery in the larvae with eye enucleation and optic nerve transection, compared to those with monocular occlusion and optic nerve transection (Fig. 4f). These findings suggest that the contralateral eye plays a critical role in the functional recovery of the optic nerve following transection.

**Fig. 5:**
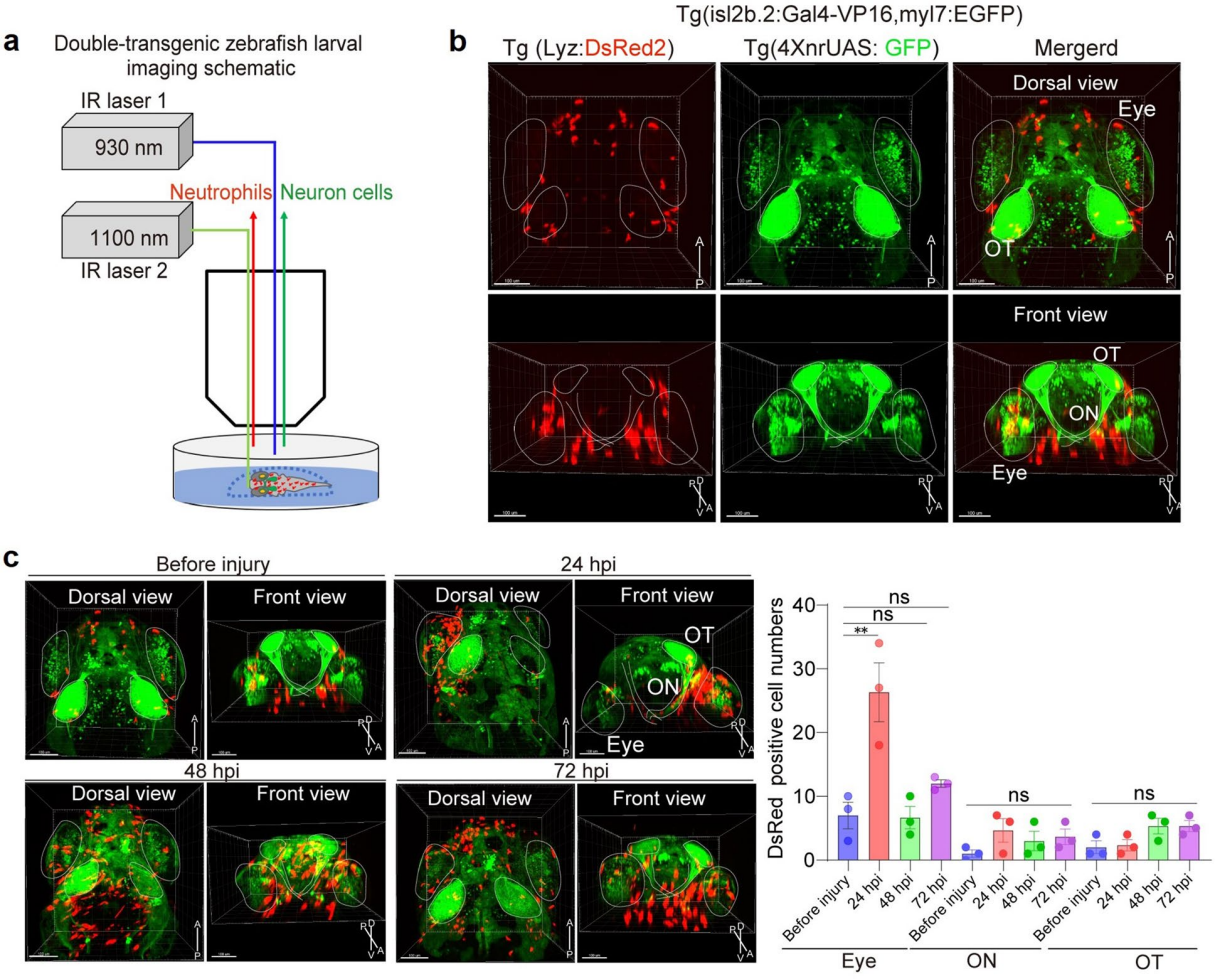
Imaging of double-transgenic zebrafish larvae before and after injury. **a** Schematic of the larval imaging workflow. **b** Upper panel: dorsal view of the double fluorescent transgenic larvae; lower panel: front view. Scale bar = 100 µm. White dotted traces indicate landmarks of the eye, optic nerve (ON) and optic tectum (OT). **c** Left panel: imaging of larvae at different time points after optic nerve transection. Scale bar = 100 µm. The anterior (A), posterior (P), dorsal (D), and ventral (V) sides are indicated. Right panel: quantification of neutrophil presence in the eye, ON and OT regions at pre-injury, 24 hpi, 48 hpi and 72 hpi time points. Sample sizes: n = 3 larvae for pre-injury, n = 3 larvae for 24 h post-injury, n = 3 larvae for 48 h post-injury and n = 3 larvae for 72 h post-injury groups. Statistical analysis was performed using one-way ANOVA with Dunnett’s test. Significance levels: ns, not significant, **P < 0.01

We further employed our two-photon imaging system to explore cell–cell interplay during the regeneration of the visual pathway after optic nerve transection. Previous studies found that neutrophils may involve optic nerve regeneration [22]. To concurrently label neutrophils and neural structures, double-transgenic zebrafish were generated by crossing *Tg(Lyz:DsRed)* [neutrophil-specific] and *Tg(isl2b.2:Gal4-VP16, myl7:EGFP); Tg(4XnrUAS:GFP*) lines. Dual-wavelength excitation (930 nm for GFP, 1,100 nm for DsRed) enabled simultaneous tracking of the optic nerve architecture and neutrophil behavior (Fig. 5a). In uninjured zebrafish larvae, neutrophils exhibited uniform distribution across the retina and optic tectum (Fig. 5b). At 24 hpi, neutrophils were predominantly localized at the injury site (Fig. 5c). As the injury progressed, their numbers in the eye gradually declined. Similarly, there was an increased presence of neutrophils in the optic nerve at 24 hpi and in the optic tectum at 48 hpi (Fig. 5c). This spatially targeted recruitment suggests that neutrophils may act as active participants in axonal repair processes during optic nerve regeneration.

## Discussion

Our study revealed a spatiotemporally coordinated relationship between the structural regeneration of the optic nerve and the functional recovery of visual behavior in zebrafish larvae, as assessed by two-photon imaging. This spatiotemporal map provides critical insight into the dynamic interplay between distinct regions of the visual pathway during regeneration, linking cellular and circuit-level repair to behavioral restoration. We investigated post-injury regenerative dynamics within a fully established neural circuit by focusing on zebrafish larvae 5 dpf, a developmental stage at which the visual system is functionally mature [23–25]. This approach is crucial for capturing the complexity of regeneration in a developed system and understanding the extensive visual field encompassing the entire visual pathway.

Previous studies have reported that PTU treatment can affect eye size and visual behavior [14, 26]. In our study, PTU use was carefully controlled, with consistent concentrations and exposure durations to minimize potential developmental effects. To assess the impact of PTU on visual function, we compared OKR responses between larvae treated with PTU and untreated controls. Larvae were exposed to PTU for 5 days, followed by a 2-day recovery period to allow partial restoration of eye pigmentation prior to OKR testing. No significant differences in OKR performance were observed between the two groups. It is also well-documented that PTU can lead to physiological changes such as reduced hatching rates and delayed development [13, 27, 28]. Though it seems that PTU treatment may delay or impair functional recovery following optic nerve transection, here we observed robust axonal regrowth following both partial and complete optic nerve transections, indicating that PTU treatment does not interfere with optic nerve regeneration.

In vivo imaging provides valuable insights into the dynamic interplay between immune cells, neurons, and glial cells during regeneration. The involvement of immune responses in tissue repair has been extensively documented [29, 30]. Our findings align with this understanding, demonstrating localized neutrophil accumulation at the site of injury, consistent with prior evidence indicating that regulated inflammation promotes tissue regeneration in zebrafish.

In zebrafish, the optic nerve exhibits a remarkable capacity for regeneration following optic nerve crush injury, a process heavily influenced by the dynamic interplay between immune and glial cells. This cellular crosstalk is essential for the coordination of key regenerative events, including axonal regrowth and remyelination. Notably, a pivotal study demonstrated that intraocular inflammation induced by zymosan significantly enhances optic nerve regeneration, highlighting the pro-regenerative role of immune activation in the central nervous system of zebrafish [31]. Furthermore, zebrafish lacking microglia exhibited accelerated axonal regrowth accompanied by atypical Müller glial proliferation, highlighting the intricate and context-dependent roles that these cell types play during regeneration [31]. This observation suggests that although microglia are critical for coordinating regenerative responses, their absence deregulates glial behavior and alters the regenerative trajectory.

In the context of remyelination, zebrafish fully restore the myelin sheaths around the optic nerve axons following demyelination. This process involves the proliferation and differentiation of oligodendrocyte progenitor cells, which successfully reestablish the myelin architecture. However, in aged zebrafish, remyelination leads to thinner and less compact myelin sheaths, a phenomenon associated with a reduced macrophage/microglial response, indicating that the efficacy of regenerative processes diminishes with age and is tightly associated with immune–glial interactions [32].

Furthermore, recent studies have reported that resident and retinal oligodendrocytes contribute to the regeneration of the zebrafish optic nerve. After injury, mature oligodendrocytes are transiently lost, creating a permissive environment for regeneration [33]. In parallel, Sox2^+^ progenitor cells proliferate and differentiate into new oligodendrocytes, supporting the remyelination of regenerated axons. This process highlights a tightly regulated sequence of cellular events in which oligodendrocyte turnover and progenitor activation are essential for restoring myelin integrity [33].

Previous studies have reported that optic nerve regeneration in zebrafish larvae occurs rapidly and independently of RGC death or proliferation, with regenerating axons successfully reinnervating appropriate areas of the optic tectum [8].

In this study, we conducted both simple linear regression analysis and multiple linear regression analysis to investigate the relationship between fluorescence recovery in the eye, optic nerve, and optic tectum with behavioral recovery. Both analyses consistently indicated that fluorescence recovery in the optic nerve and optic tectum are key predictors of behavioral recovery, whereas fluorescence recovery in the eye showed no significant association. Furthermore, for multiple linear regression analysis, the sample size should be at least 5 to 10 times the number of independent variables. Given the limited number of surviving fish after injury in our current analysis, future studies will need to increase the sample size and analyze other factors, such as PTU treatment.

Our study advances the understanding of optic nerve regeneration by highlighting the critical role of the contralateral eye in functional recovery after optic nerve transection.

Previous studies have shown that complete bilateral transection of the optic nerves leads to more pronounced aberrant axonal projections compared to partial or unilateral transection, although the functional significance of these projections remains unclear, and optic nerve regeneration is not strictly tectum-specific, even when the contralateral eye is intact [8]. However, the direct relationship between these anatomical alterations and functional restoration has remained unclear. Here, by employing a model that integrates unilateral enucleation with contralateral optic nerve transection, we observed extensive aberrant axonal projections and, crucially, demonstrated for the first time that the absence of the contralateral eye severely impedes functional recovery as assessed by OKR. These results reveal a previously unrecognized yet critical role for the contralateral eye in facilitating successful functional reconnection between regenerating optic nerve fibers and the brain. Although the molecular mechanisms through which unilateral enucleation disrupts functional recovery of the contralateral eye after optic nerve injury remain to be elucidated, such impairment likely involves contralateral modulatory effects within the central nervous system (CNS). Unilateral injury can trigger inflammatory responses and neuronal degeneration on the uninjured side, potentially influencing the recovery process [34]. Future studies, including spatial transcriptomic approaches, will be important for delineating the molecular guidance mechanisms that facilitate successful axonal regeneration and functional recovery [35].

Previous studies have indicated that optic tectum ablation in zebrafish preserves OKR but reduces saccade frequency, whereas tracking velocities, gain, and saccade amplitude remain unchanged [24]. This suggests that the tectum does not play a central role in extracting higher-order motion cues within the visual pathway, highlighting the complexity of functional recovery. High-resolution imaging at the cellular level was instrumental in capturing these detailed regenerative processes, allowing us to observe axon regrowth and synaptic reformation with precision.

By combining structural and functional analyses, this study advances our understanding of neural repair mechanisms in zebrafish and underscores the utility of this model for uncovering evolutionarily conserved pathways with potential therapeutic relevance for CNS injuries in mammals. Our findings also emphasize the importance of determining whether functional connectivity develops between the transplanted eye and the optic nerve, and whether regenerating optic nerve fibers accurately project to their appropriate brain targets.

## Limitations and future directions

Although this study delineated the early phases of circuit regeneration, longitudinal assessments spanning functional refinement and synaptic reorganization are required to determine whether the restored networks recapitulate pre-injury computational fidelity. Furthermore, our characterization of visually guided behaviors remains restricted to the OKR—a foundational stabilization reflex [10]. Mechanistic insights into vision related to ethological critical processes, such as predation and phototaxis, remain unresolved [36, 37]. Resolving these questions requires a systematic dissection of how distributed neural ensembles encode stimulus valence, integrate internal states, and execute adaptive motor plans, ideally through cross-species comparisons and closed-loop virtual reality paradigms. A synergistic approach combining single-cell spatiotemporal transcriptomics, in vivo calcium imaging, and optogenetic perturbation could decode the instructive cues governing regenerative precision [38–43]. Such efforts may elucidate the connection between neural repair and the plasticity of behaviorally relevant neural circuits.

## Conclusion

Our optimized imaging platform allows high-resolution, comprehensive visualization of the entire visual pathway and cell–cell interactions during regeneration in larval zebrafish following optic nerve transection. Using this system, we demonstrate that the contralateral eye plays a crucial role in visual recovery following optic nerve transection. The optimized two-photon in vivo imaging, together with spatial transcriptomics and single-cell transcriptomics, provides a powerful platform for future studies aimed at deciphering the cellular and molecular mechanisms underlying the functional reconstruction of the eye-brain pathway in zebrafish, and offer valuable insights for the development of therapeutic targets for optic neuropathies in humans.

## Supplementary Information

Supplementary material 1: Table S1. Multiple linear regression analysis of functional recovery with eye and optic nerve fluorescence recovery. Table S2. Multiple linear regression analysis of functional recovery with eye and optic tectum fluorescence recovery. Table S3. Multiple linear regression analysis of functional recovery with optic nerve and optic tectum fluorescence recovery.

## Data Availability

The datasets supporting the conclusions of this article are included within the article. Supplementary data to this article can be found online.

## Abbreviations

PTU: Phenylthiourea
RGC: Retinal ganglion cell
DPF: Days post fertilization
OKR: Optokinetic response
CNS: Central nervous system
