# Supplementary material for: Optimized in vivo two-photon imaging reveals the essential role of the contralateral eye in functional optic nerve regeneration in zebrafish larvae
Source: Eye Vis (Lond). 2025 Aug 25;12:34. doi: 10.1186/s40662-025-00447-z (PMC12376317; doi:10.1186/s40662-025-00447-z)
Supplement: Supplementary file 1 — Supplementary material 1: Table S1. Multiple linear regression analysis of functional recovery with eye and optic nerve fluorescence recovery. Table S2. Multiple linear regression analysis of functional recovery with eye and optic tectum fluorescence recovery. Table S3. Multiple linear regression analysis of functional recovery with optic nerve and optic tectum fluorescence recovery. [file 40662_2025_447_MOESM1_ESM.docx]

**Supplementary Table S1.** Multiple linear regression analysis of functional recovery with eye and optic nerve fluorescence recovery.

| **Variables** | **β** | **95% CI** | | **VIF** | ***P*** |
| --- | --- | --- | --- | --- | --- |
|  |  | **Lower bound** | **Upper bound** |  |  |
| Eye | −0.114 | −0.374 | 0.145 | 1.000 | ns |
| Optic nerve (ON) | 0.274 | 0.172 | 0.376 | 1.000 | 0.0006 |
| Dependent variable: number of eye movements | | | | | |
| R: 0.938, R Square: 0.881, Adjusted R Square: 0.841 | | | | | |

Multiple linear regression equation: number of eye movements = −0.114*eye + 0.274*ON − 0.107, *P* = 0.0017.

The β (estimate [95% CI]) indicates the size and direction of each variable's effect on the dependent variable. CI = confidence interval; VIF = variance inflation factor; ns = not significant

**Supplementary Table S2.** Multiple linear regression analysis of functional recovery with eye and optic tectum fluorescence recovery.

| **Variables** | **β** | **95% CI** | | | **VIF** | ***P*** |
| --- | --- | --- | --- | --- | --- | --- |
|  |  | **Lower bound** | **Upper bound** | |  |  |
| Eye | −0.063 | −0.404 | | 0.279 | 1.006 | ns |
| Optic tectum (OT) | 0.168 | 0.081 | | 0.255 | 1.006 | 0.0032 |
| Dependent variable: number of eye movements | | | | | | |
| R: 0.891, R Square: 0.794, Adjusted R Square: 0.726 | | | | | | |

Multiple linear regression equation: number of eye movements = −0.063*eye + 0.168*OT + 0.123, *P* = 0.0087.

The β (estimate [95% CI]) indicates the size and direction of each variable's effect on the dependent variable. CI = confidence interval; VIF = variance inflation factor; ns = not significant

**Supplementary Table S3.** Multiple linear regression analysis of functional recovery with optic nerve and optic tectum fluorescence recovery.

| **Variables** | **β** | **95% CI** | | **VIF** | ***P*** |
| --- | --- | --- | --- | --- | --- |
|  |  | **Lower bound** | **Upper bound** |  |  |
| Optic nerve (ON) | 0.187 | −0.152 | 0.389 | 4.087 | ns |
| Optic tectum (OT) | 0.064 | −0.066 | 0.195 | 4.087 | ns |
| Dependent variable: number of eye movements | | | | | |
| R: 0.94, R Square: 0.885, Adjusted R Square: 0.847 | | | | | |

Multiple linear regression equation: number of eye movements = 0.187*ON + 0.064*OT − 3.471, *P* = 0.0015.

The β (estimate [95% CI]) indicates the size and direction of each variable's effect on the dependent variable. CI = confidence interval; VIF = variance inflation factor; ns = not significant
